# INTOLERANCE FOR UNCERTAINTY MEDIATES DEATH ANXIETY AND HYPOCHONDRIASIS IN OLDER ADULTS

**DOI:** 10.1093/geroni/igad104.3014

**Published:** 2023-12-21

**Authors:** Derek Killingsworth, Joseph Muraira, Matthew Fontanese, Hannah Cornwell, Lance Barela, Karina Kapoor, Allyson Coldiron, Michael Barnett

**Affiliations:** University of Texas at Tyler, Tyler, Texas, United States; University of Texas at Tyler, Tyler, Texas, United States; University of Texas at Tyler, Tyler, Texas, United States; University of Texas at Tyler, Tyler, Texas, United States; University of Texas at Tyler, Tyler, Texas, United States; University of Texas at Tyler, Tyler, Texas, United States; University of Texas at Tyler, Tyler, Texas, United States; University of Texas at Tyler, Tyler, Texas, United States

## Abstract

Hypochondriasis – or illness anxiety disorder – is the preoccupation of having an illness, which has been linked with and theorized to be caused by fear of death. While death anxiety tends to decrease with age because of increased exposure to death, the association between illness anxiety and fear of death strengthens with age. Many anxiety disorders, including illness anxiety disorder, have often been explained by an intolerance for uncertainty. The current study therefore investigated whether intolerance of uncertainty mediated the effect of fear of death on hypochondriasis among older adults. Older adults (N = 260), ages 65 to 94 years (M = 75.8, SD = 7.39), responded to an online survey, which included the Death Attitude Profile-Revised questionnaire, the Intolerance of Uncertainty Scale, and the Illness Attitudes Scale. Results showed that fear of death was directly related to hypochondriasis as well as indirectly related through intolerance of uncertainty. Fear of death and intolerance of uncertainty were both positively associated with hypochondriasis. Older adults with more fear about death are more likely to experience illness anxiety and have a higher intolerance for uncertainty. It may be that older adults with higher levels of intolerance to uncertainty are more at risk for anxiety, which then may result in increased fear of death and illness anxiety with the increased exposure to death that is associated with older age. Hypochondriasis in older adulthood may therefore be best addressed by interventions that explore the uncertainty of death and focus on increasing tolerance for uncertainty.

